# Comparing Internet Assistance for Smoking Cessation: 13-Month Follow-Up of a Six-Arm Randomized Controlled Trial

**DOI:** 10.2196/jmir.1008

**Published:** 2008-11-21

**Authors:** Vance Rabius, K Joanne Pike, Dawn Wiatrek, Alfred L McAlister

**Affiliations:** ^2^University of Texas Health Science CenterHoustonTXUSA; ^1^American Cancer SocietyAustinTXUSA

**Keywords:** Smoking cessation, Internet, cigarette smoking, randomized controlled trial

## Abstract

**Background:**

Although many smokers seek Internet-based cessation assistance, few studies have experimentally evaluated long-term cessation rates among cigarette smokers who receive Internet assistance in quitting.

**Objective:**

The purpose of this study is to describe long-term smoking cessation rates associated with 6 different Internet-based cessation services and the variation among them, to test the hypothesis that interactive and tailored Internet services yield higher long-term quit rates than more static Web-posted assistance, and to explore the possible effects of level of site utilization and a self-reported indicator of depression on long-term cessation rates.

**Method:**

In 2004-05, a link was placed on the American Cancer Society (ACS) website for smokers who wanted help in quitting via the Internet. The link led smokers to the QuitLink study website, where they could answer eligibility questions, provide informed consent, and complete the baseline survey. Enrolled participants were randomly assigned to receive emailed access to one of five tailored interactive sites provided by cooperating research partners or to a targeted, minimally interactive ACS site with text, photographs, and graphics providing stage-based quitting advice and peer modeling.

**Results:**

6451 of the visitors met eligibility requirements and completed consent procedures and the baseline survey. All of these smokers were randomly assigned to one of the six experimental groups. Follow-up surveys done online and via telephone interviews at approximately 13 months after randomization yielded 2468 respondents (38%) and found no significant overall quit rate differences among those assigned to the different websites (*P* = .15). At baseline, 1961 participants (30%) reported an indicator of depression. Post hoc analyses found that this group had significantly lower 13-month quit rates than those who did not report the indicator (all enrolled, 8% vs 12%, *P* < .001; followed only, 25% vs 31%, *P* = .003). When the 4490 participants (70%) who did not report an indicator of depression at baseline were separated for analysis, the more interactive, tailored sites, as a whole, were associated with higher quitting rates than the less interactive ACS site: 13% vs 10% (*P* = .04) among 4490 enrolled and 32% vs 26% (*P* = .06) among 1798 followed.

**Conclusions:**

These findings show that Internet assistance is attractive and potentially cost-effective and suggest that tailored, interactive websites may help cigarette smokers who do not report an indicator of depression at baseline to quit and maintain cessation.

## Introduction

To help serve the millions of smokers who can reduce their cancer risk by quitting, the American Cancer Society (ACS) offers telephone counseling and other services. One randomized trial found telephone counseling provided by the ACS to cost US $1300 per long-term quitter [1], well below the health-related costs of tobacco use of US $3391 per smoker per year estimated by the Centers for Disease Control and Prevention [2]. The ACS call center for smokers has grown to serve approximately 70,000 persons in 2006 at a modest cost per client. However, Internet service provides greater potential for cost-efficiency. With scalability, Internet services can provide assistance to many smokers at a very reasonable cost.

Although there are hundreds of websites that provide information about smoking cessation, fewer than one in 20 provide assistance that meets the basic standards regarding the use of evidence-based content of cessation advice, and very few provide useful interactivity [3]. Studies of websites that do provide interactive tailoring with evidence-based instruction and peer modeling have shown promising results [4], although it is difficult to obtain sustained use of online assistance as smokers attempt to go through the quitting process [5]. Randomized studies have found significant effects on sustained quitting for approximately 3 to 7 months from interactively tailored sites with regular out-bound emailed queries and messages designed to help smokers take discrete steps toward long-term cessation [6,7].

Internet-based assistance can offer both interactively tailored advice and online communities that allow visitors to learn from one another and receive social support. Reporting on a website with chat rooms, Cobb et al [8] found that 3-month maintained quitting rates among visitors were highly related to how many times the chat rooms were visited. In the short-term findings from the research reported here, in which diverse interactive sites were compared, the sites that obtained the highest number of visits produced the highest rates of cessation at 4 months following registration [9]. No large-scale randomized trials to rigorously assess the longer term effects of Internet assistance have previously been reported.

Depression is an important factor in smoking cessation [10]. There are generally high levels of depression among smokers [11], and depression inhibits quitting success by decreasing self-efficacy [12]. In research on smokers seeking assistance from telephone counseling, high levels of self-reported depression have been observed. Those with a valid, single-item [13] depression indicator were found to achieve lower rates of sustained cessation that those without it [14]. In that study, telephone counseling had a modest effect on those with the indicator, while its strongest effect was among those without it. In a more recent study that measured the frequency of self-reported depression on an interval scale [15], approximately half of the clients reported depression symptoms in response to the single-item indicator. In that study, enhancements in the protocol using cognitive therapy for depression did not significantly increase the quit rate among those with an indication of depression.

The present study was designed to describe long-term effects on quit rates among smokers using the Internet for quitting assistance and compare the 13-month follow-up quit rates for visitors to tailored interactive sites with the quit rate for visitors to a targeted, relatively static site provided by the ACS. Five interactive sites, each with somewhat different features, were selected to explore possible variations in their effectiveness. The planned hypotheses to be tested were that (1) quit rates will differ between sites and (2) tailored, interactive sites will have a greater effect than the targeted, relatively static site. Exploratory analyses were conducted to determine if quitting success is linked to the number of visits to the interactive sites. An additional post hoc analysis tested the hypothesis that quit rates for more interactive sites were greater among those who did not report an indicator of depression than among those who did.

## Methods

A link was placed on the American Cancer Society (ACS) website for smokers who wanted help in quitting via the Internet. The link led smokers to the QuitLink study website, where they could answer eligibility questions, provide informed consent, and complete the baseline survey. The enrolled participants were randomly assigned to receive emailed access to one of five tailored interactive sites provided by cooperating research partners or to a targeted, minimally interactive ACS site with text, photographs, and graphics providing stage-based quitting advice and peer modeling.

 The five research partners (Table 1) agreed to provide study participants with access to their Internet smoking cessation services free of charge. A targeted, relatively static site containing evidence-based self-help information and peer modeling (provided in print form for smokers using the ACS telephone counseling) was also posted on the Internet for use in this study. Participants entered the study via self-referral through the main ACS website, which is widely promoted in ongoing public communication. They enrolled by clicking a link to receive information about the project and completing a human subject consent protocol and baseline survey with a further link leading to randomization. They were then emailed a link to one of the six Internet sites listed in Table 1. This provided immediate access at no cost. Of course, participants could, for a fee, subsequently enroll in these or other Internet cessation services.

**Table 1:** Research sites and characteristics

Site	Description
Oregon Center for Applied Sciences	Included presentations with role models that mimic a counseling experience
ProChange	Featured extensive stage-based tailoring
QuitNet	Featured an online “community” with chat rooms
SmokeClinic	Included mood assessment and chat rooms
CAMH by V-CC	Included online community features and instant messaging
ACS – Break Away from the Pack PDF files	Included text, photographs, and graphics with stage-based peer modeling

Participation in the study was limited to English-speaking daily smokers residing in the United States who provided informed consent and completed the baseline survey. Enrollment was conducted from October 2004 through May of 2005, with a goal of 1000 participants per site and half that number at follow-up to provide sufficient statistical power for detecting meaningful differences between quit rates in the different experimental groups. Baseline data included demographics, smoking history, and questions about Internet use. A single-item depression indicator [13] asked participants whether or not they felt “sad or blue” every day for the past 2 weeks. Another question asked participants to rate their self-efficacy for quitting on 0-100 scale, with 0 corresponding to no confidence and 100 corresponding to complete confidence in being able to quit and maintain cessation. Data were collected from each of the five site providers on registration and the number of visits to the site by registered participants.

Follow-up surveys were conducted approximately 4 months and then 13 months after randomization, first by email and then by telephone to reach those who did not respond to the email inquiry. The quit rate indicator that was selected for analysis was successful abstinence for 30 days prior to the follow-up contact (30-day point prevalence). This is a standard criterion for assessing cessation in studies of telephone counseling [16], which allows for the possibility of slips or brief relapses during the months preceding the follow-up interview.

As fewer than half of the participants provided follow-up data, 13-month quit rates were calculated both as the proportion among followed participants and as the proportion among all enrolled participants—assuming that those who did not provide follow-up data did not quit smoking (intent-to-treat analysis). In the tests of hypotheses about variation between sites and in exploratory and post hoc analyses, chi-square statistics were used to calculate the significance of observed differences in the 13-month quit rates. These involved 2 × 6 (quitting status by randomized group), 2 × 4 (quitting status by grouped number of visits), and 2 × 2 (quitting status by pooled or selected groups) chi-square tabulations in the different significance tests that were performed.

This research was approved (HSC-SPH-04-038) by the University of Texas Health Science Center at Houston Committee for Protection of Human Subjects. The trial was not registered, because enrollment started before trial registration became mandatory.

## Results

From October 2004 to May 2005, there were over 7 million visitors to the main page of the ACS website and 241,223 visitors to the part of the site concerned with smoking cessation, where the project invitation appeared. There were 44,616 visitors to the project entry page, but only 6451 of these visitors met eligibility requirements and completed consent procedures and the baseline survey. All of these smokers were randomly assigned to one of the six experimental groups. Participants were mostly women (70%), with a mean age of 41 years, a mean smoking rate of 21 cigarettes per day, and an average of 6.3 previous quit attempts. These features are similar to those of smokers who register to use the ACS telephone service to assist smoking cessation [1]. However, Internet study participants were more educated (75% vs 59% receiving some college education), more likely to be Caucasian (87% vs 74%), and less likely to report an indicator of depression when compared to smokers participating in our previous studies of telephone counseling (30% vs 40% or more in different studies). Multiple daily Internet use was reported by 66% of participants, and 21% reported using the Internet once a day. As Table 2 illustrates, there were no important baseline differences between the six experimental groups.

**Table 2:** Participant baseline characteristics by group ^a,b^

Characteristic	Control Site (n = 1047)	Site 1 (n = 1052)	Site 2 (n = 1103)	Site 3 (n = 1042)	Site 4 (n = 1101)	Site 5 (n = 1106)
**Gender**						
Women	728 (70)	754 (72)	791 (72)	753 (72)	796 (72)	764 (69)
Men	319 (30)	298 (28)	312 (28)	289 (28)	305 (28)	342 (31)
**Ethnicity**						
White	920 (88)	926 (88)	972 (88)	902 (87)	954 (87)	968 (88)
**Education**						
Elementary school	5 (0)	5 (0)	2 (0)	4 (0)	5 (0)	6 (1)
Some high school	28 (3)	21 (2)	34 (3)	34 (3)	34 (3)	38 (3)
High school graduate	186 (18)	205 (19)	214 (19)	218 (21)	244 (22)	223 (20)
Some college	545 (52)	504 (48)	531 (48)	484 (46)	501 (46)	534 (48)
College graduate	277 (26)	312 (30)	316 (29)	301 (29)	306 (28)	298 (27)
Refused to answer	6 (1)	5 (0)	6 (1)	1 (0)	11 (1)	7 (1)
**Living Situation**						
Alone	187 (18)	193 (18)	209 (19)	186 (18)	193 (18)	194 (18)
With a nonsmoker	447 (43)	437 (42)	438 (40)	458 (44)	495 (45)	490 (44)
With a smoker	413 (39)	422 (40)	455 (41)	368 (38)	413 (38)	422 (38)
**Current Mood**						
Sad or blue	313 (30)	314 (30)	354 (32)	296 (28)	351 (32)	333 (30)
**Internet Use**						
Less than once a week	11 (1)	11 (1)	12 (1)	13 (1)	12 (1)	14 (1)
Once a week	16 (2)	24 (2)	20 (2)	20 (2)	15 (1)	19 (2)
Twice a week	36 (3)	26 (2)	36 (3)	37 (4)	32 (3)	26 (2)
Every other day	80 (8)	83 (8)	81 (7)	62 (6)	77 (7)	77 (7)
Once a day	225 (21)	218 (21)	217 (20)	208 (20)	238 (22)	247 (22)
Several times a day	679 (65)	690 (66)	737 (67)	702 (67)	727 (66)	723 (65)
Self-efficacy, mean (SD)	70.4 (23.4)	71.6 (23.0)	71.9 (22.8)	71.3 (22.6)	70.8 (23.8)	71.3 (24.1)
Age (years), mean (SD)	40.8 (11.1)	40.6 (11.3)	40.5 (11.4)	40.6 (10.9)	40.7 (11.3)	40.5 (11.3)
**Daily Cigarette Use**						
Weekday, mean (SD)	20.2 (12.3)	19.4 (11.2)	19.2 (10.6)	19.1 (10.4)	19.9 (12.0)	19.3 (11.3)
Weekend, mean (SD)	22.6 (11.3)	21.9 (11.0)	22.0 (11.0)	21.9 (11.2)	22.0 (11.5)	21.5 (11.1)
Previous quit attempts, mean (SD)	5.3 (5.7)	5.3 (6.8)	5.3 (6.6)	5.4 (6.7)	5.5 (6.5)	5.2 (5.4)
Number of years smoking, mean (SD)	22.0 (11.4)	21.7 (11.3)	21.7 (11.4)	21.6 (11.2)	21.9 (11.3)	21.8 (11.4)

^a^ Values are expressed as No. (%) unless otherwise indicated.

^b^ The order of the sites does not correspond to Table 1.

Of the 6451 clients enrolled in the study, only 2468 (38%) provided information on their smoking status 13 months after randomization. The follow-up rates did not differ significantly between the six experimental groups. Quit rates, whether calculated as a proportion of those followed or of those enrolled, also did not differ significantly between the groups, failing to confirm our first hypothesis about variation in effectiveness. The second main hypothesis was also not confirmed, as there was no overall difference in 13-month quit rates between the entire group of smokers assigned to any of the five interactive sites and those assigned to the static ACS site. Approximately 10% of enrolled participants reported sustained cessation at that time point among those assigned to the static site, with rates ranging from approximately 8% to 12% among those assigned to the five different interactive sites. Calculated less conservatively as a proportion of those who were followed, the 13-month cessation rates ranged from approximately 20% to 30% among those in the five interactive groups.

**Figure 1:**
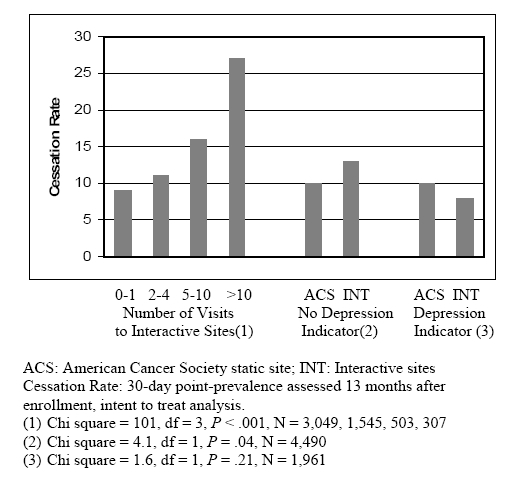
13-month quit rates by number of visits, indicator of depression, and type of site, either interactive (INT) or static ACS site (cessation rate is 30-day point prevalence assessed 13 months after enrollment; intent-to-treat analysis assumes dropouts did not quit

Exploratory analyses, which arbitrarily grouped participants according to their level of exposure to the interactive sites, found a very strong observed relationship between quit rates after 13 months (as a proportion of all enrolled participants) and the number of times participants visited the site (see Figure 1). Most participants did not make more than two visits, and only 810 visited five times or more. However, the 13-month quit rate among those visiting five times or more was two times higher than among those with fewer than five visits (20.0 vs 9.8, *P* < .001). Short-term results published previously from this research also showed a significant difference in 4-month quit rates among the tailored, interactive sites when sites were similarly grouped by level of utilization [9]. That finding was replicated in exploratory analyses of these 13-month follow-up data, with higher quit rates associated with the two most highly utilized sites than with the three less frequently utilized sites (intent-to-treat, 12.5 vs 10.6, *P* = .03; respondent only, 32.1 vs 27.9, *P* = .04).

A total of 1961 study participants (30%) reported at baseline feeling “sad or blue” every day for the last 2 weeks, which provided an indication of self-reported depression. Post hoc analyses found that this group had significantly lower quit rates (intent-to-treat, 8.4 vs 12.3, *P* < .001; respondent only, 24.6 vs 30.8, *P* = .003). Groups were analyzed separately, based on the response to the depression indicator, to examine differential Internet assistance effects in those two groups. These post hoc chi-square analyses found that participants who did not report the depression indicator and were referred to the more interactive sites reported higher 13-month quit rates than those referred to the less interactive ACS site. As shown in Figure 1, the respective quit rates were 13% and 10% (*P* = .04) among 4490 enrolled participants not reporting the indicator of depression in an “intent-to-treat” estimation of experimental effects. Interestingly, further post hoc analyses found that participants with the depression indicator had significantly lower 13-month quit rates than those without it if they were assigned to interactive sites (8% vs 13% among all enrolled 5404 participants, *P* < .001), but not if they were referred to the static ACS site (10% for those with or without the depression indicator among 1047 enrolled participants, *P* = .94).

## Discussion

This study was exploratory and was not designed to rigorously test specific components of Internet assistance for quitting smoking but rather to describe and compare the long-term effectiveness of currently available programs. It provides self-reported data on cessation status without verification. While the follow-up response rate was only 38%, previous studies with monetary incentives for Internet research participants yielded responses from only about half of the study participants [4]. As Eysenbach [17,18] has noted, research on this topic is inevitably flawed by attrition. The intent-to-treat standard for assessing quit rates as a proportion of enrolled rather than followed participants may bias research on Internet-based cessation services toward the null hypothesis of no treatment effect. Enrollment in an Internet service should be deliberately designed to make it easy for the user, but the impersonal nature of Internet enrollment also makes it possible for people to enroll without making any real commitments to what they have signed up to do. This feature probably also explains the generally observed difficulty with continuation rates in Internet assistance (eg, [5]) and the low continuation rates observed here.

Although no significant differences were observed between the quit rates of different tailored, interactive sites in this 13-month follow-up, it is possible that differences were not detected because sample sizes in this study did not provide the statistical power for tests of differences of less than approximately 3% to 6% among the enrolled and followed groups, respectively. Among participants without an indicator of depression, the significantly different 13-month quit rates in the tailored, interactive sites and the targeted, relatively static site (13% vs 10% of enrolled and 32% vs 36% of followed participants) compare favorably with the long-term quit rates typically reported by telephone counseling interventions [1,19,20].

No similarly long-term study of Internet quitting assistance has been previously reported, and, despite the unavoidable limitations of unvalidated self-reports of cessation and a high rate of loss to follow-up, this exploratory study allows some cautious conclusions. It shows that Internet assistance is attractive and cost-effective. In a relatively short time, more than 6000 users enrolled through the link posted on the regularly publicized ACS website. Service was provided with no costs other than those associated with establishment of the website linkages and the targeted, relatively static site posting. Approximately 4 days of programming at a cost of less than US $2000 allowed approximately 5000 additional users for scalable services from the five tailored, interactive service providers. This contrasts with the much larger cost of serving 1000 new clients with telephone counseling (approximately US $100,000).

Based on previous studies of telephone counseling showing lower cessation effects among clients with an indicator of depression than among those without [14,15], it is not surprising to find a similar result in post hoc analyses of the data from this study. Studies that do demonstrate long-term intervention effects on cessation among depressed smokers involve much more intensive personal contact in individualized learning sessions (eg, [21]).

It was surprising to find that those who reported the depression indicator and were assigned to the tailored, interactive sites had, although not significantly, lower cessation rates than those assigned to the targeted, relatively static site. One major difference between the tailored, interactive sites and the targeted, relatively static site was the time that the participant must invest in the site. The tailored, interactive sites required the participant to spend time registering and providing the personal details that inform the tailoring, whereas the targeted, relatively static site was accessed without registration. The participant could link to the site and immediately read or download the materials for later reference. The greater effort required to participate in the interactive sites may have acted as a deterrent to quitting among depressed smokers.

Short-term results from the present study [9] found that cessation rates were strongly related to the mean number of visits to the different sites. Those sites with the most visits tended to produce better short-term results. This finding was repeated in the post hoc analyses reported here, which also show a strong relationship between number of visits and long-term quitting success. Future studies should employ randomized experimental designs to rigorously examine the effectiveness of various discrete features of Internet assistance, such as outbound emails, chat rooms, and other ways of engaging users and providing support for the quitting process.

The possible influence of depression on the effectiveness of interactive Internet assistance requires further research with more complex indicators of depression to examine how they relate to specific processes and responses in Internet-assisted smoking cessation. In this study, about one in three participants reported an indicator of depression, which is lower than the one in two rate that we currently find among research participants receiving telephone counseling [15]. However, this rate is still substantial, and we hope in further studies to identify ways to effectively provide everyone who seeks help via the ACS main website with services that can increase their odds of quitting.

The present study suggests that a high volume of use may be expected for Internet sites that offer useful assistance for smoking cessation. However, many persons seeking to quit smoking do not choose the Internet for assistance. The present study suggests that Internet-based cessation assistance may appeal to clients who are much more educated and presumably more comfortable with Internet communication than those who seek telephone-based assistance. Both modalities of service should be provided in comprehensive smoking cessation assistance programs.

## Abbreviations

ACS: American Cancer Society
